# Uptake and retention on HIV pre‐exposure prophylaxis among key and priority populations in South‐Central Uganda

**DOI:** 10.1002/jia2.25588

**Published:** 2020-08-12

**Authors:** Joseph Kagaayi, James Batte, Hadijja Nakawooya, Boniface Kigozi, Gertrude Nakigozi, Susanne Strömdahl, Anna Mia Ekström, Larry W Chang, Ron Gray, Steven J Reynolds, Patrick Komaketch, Stella Alamo, David Serwadda

**Affiliations:** ^1^ Rakai Health Sciences Program Kalisizo Uganda; ^2^ Makerere University School of Public Health Kampala Uganda; ^3^ Johns Hopkins Bloomberg School of Public Health Baltimore MD USA; ^4^ Johns Hopkins School of Medicine Baltimore MD USA; ^5^ Department of Global Public Health and Karolinska University Hospital Department of Infectious Diseases Karolinska Institutet Stockholm Sweden; ^6^ Division of Intramural Research NIAID/NIH Bethesda MD USA; ^7^ Centers for Disease Control and Prevention Kampala Uganda; ^8^ Department of Medical Sciences Section of Infectious Diseases Uppsala University Stockholm Sweden

**Keywords:** PrEP, retention, HIV prevention, sex workers, risk factors, LMIC

## Abstract

**Introduction:**

Pre‐exposure prophylaxis (PrEP) programmes have been initiated in sub‐Saharan Africa to prevent HIV acquisition in key populations at increased risk. However, data on PrEP uptake and retention in high‐risk African communities are limited. We evaluated PrEP uptake and retention in HIV hyperendemic fishing villages and trading centres in south‐central Uganda between April 2018 and March 2019.

**Methods:**

PrEP eligibility was assessed using a national risk screening tool. Programme data were used to evaluate uptake and retention over 12 months. Multivariable modified Poisson regression estimated adjusted prevalence ratios (aPR) and 95% Confidence intervals (CIs) of uptake associated with covariates. We used Kaplan–Meier analysis to estimate retention and multivariable Cox regression to estimate adjusted relative hazards (aRH) and 95% CIs of discontinuation associated with covariates.

**Results and discussion:**

Of the 2985 HIV‐negative individuals screened; 2750 (92.1 %) were eligible; of whom 2,536 (92.2%) accepted PrEP. Male (aPR = 0.91, 95% CI = 0.85 to 0.97) and female (aPR = 0.85, 95% CI = 0.77 to 0.94) fisher folk were less likely to accept compared to HIV‐discordant couples. Median retention was 45.4 days for both men and women, whereas retention was higher among women (log rank, *p* < 0.001) overall. PrEP discontinuation was higher among female sex workers (aRH = 1.42, 95% CI = 1.09 to 1.83) and female fisher folk (aRH = 1.99, 95% CI = 1.46 to 2.72), compared to women in discordant couples. Male fisher folk (aRH = 1.37, 95% CI = 1.07 to 1.76) and male truck drivers (aRH = 1.49, 95% CI = 1.14 to 1.94) were more likely to discontinue compared to men in discordant couples. Women 30 to 34 years tended to have lower discontinuation rates compared to adolescents 15 to 19 years (RH = 0.78 [95% CI = 0.63 to 0.96]).

**Conclusions:**

PrEP uptake was high, but retention was very low especially among those at the highest risk of HIV: fisher folk, sex workers and truck drivers and adolescent girls. Research on reasons for PrEP discontinuation could help optimize retention.

## 1 INTRODUCTION

Pre‐exposure prophylaxis (PrEP) can help prevent HIV among individuals with substantial risk [1, 2, 3]. Studies of populations with high HIV risk in sub‐Saharan Africa (SSA) including sex workers, fisher folk and discordant couples reported 60‐90 percent willingness to use PrEP [4, 5, 6, 7, 8] but subsequent demonstration projects found mixed results for PrEP uptake: high uptake (approximately 97%), retention (>90% by three months) and adherence (over 80%) were shown among HIV‐discordant couples [9, 10] and men who have sex with men (MSMs) in Kenya [11]; whereas low uptake (approximately 18%) was observed in the Sustainable East Africa Research in Community Health (SEARCH) study [12].

Efforts to scale‐up PrEP in sub‐Saharan African countries through national health systems, require tracking of uptake, adherence and retention in PrEP programmes. We evaluated PrEP uptake and retention in a programme implemented through government clinics in districts of South‐central Uganda among individuals with high risk of HIV according to the Ugandan national HIV‐risk categorization [13].

## 2 METHODS

### 2.1 The PrEP programme

In 2017, PrEP (oral tenofovir disoproxil fumarate [TDF] and lamivudine [3TC]), was initiated in HIV hyperendemic fishing communities on Lake Victoria and trading centres in the south‐central districts including Rakai, Kyotera, Masaka and Lyantonde. This PrEP programme was implemented by the Rakai Health Sciences Program with support from the U.S. President’s Emergency Plan for AIDS Relief (PEPFAR) through the US Centers for Disease Control and Prevention (CDC) – Uganda. The programme enrolled HIV‐negative individuals with substantial HIV risk as determined by a risk screening tool developed collaboratively by the Uganda National AIDS Control Program, CDC‐Uganda and ICAP Columbia University in alignment with national PrEP guidelines [13]. Components of the risk assessment tool included the following: (1) vaginal sexual intercourse with more than one partner of unknown HIV status in the past six months; (2) vaginal sex without a condom in the past six months; (3) anal sexual intercourse in the past six months; (4) sex in exchange for money, goods or a service in the last six months; (5) Injecting drugs in the past six months; (6) diagnosis with an STI more than once in the past twelve months; (7) post‐exposure prophylaxis (PEP) for sexual exposure to HIV in the past six months; and (8) having an HIV‐infected sexual partner who was not on ART. Individuals were deemed to be at substantial risk if they reported at least one of the eight high‐risk sexual behaviours on the tool. Target groups included fisher folk, sex workers, truck drivers, HIV‐negative individuals in HIV‐discordant relationships and other individuals aged ≥15 years with substantial HIV risk including men who have sex with men (MSM) and adolescent girls and young women (15 to 24 years). Risk categories were mutually exclusive: Individuals who belonged to more than one category were classified in the dominant category where they spent most of their time. The other category included individuals with high‐risk behaviours, as indicated on the MoH assessment tool, who did not belong to any of the designated high‐risk categories.

Clients were screened and enrolled at two central facilities and eight outreach sites following community‐wide mobilization and sensitization of community leaders, health workers and special groups including sex workers, fisher folks, MSM and truck drivers. Community‐wide sensitization used messages via a megaphones in fishing communities. To minimize stigma, community‐wide mobilization only mentioned the availability of PrEP for persons at substantial risk of HIV, but did not mention any specific categories. Individuals were referred to health facilities for screening and services. Peer leaders organized sex workers and fisher folk into groups at community outreach sites for sensitization (including detailed discussions of substantial risk), screening, HTS and initiation of PrEP. Outreaches were also organized for trucker drivers at truck stops. Discordant couples received information about PrEP through HIV couples counselling sessions at health facilities. At each facility and outreach sites, services were offered by an HIV counsellor and a laboratory technician who supported HTS, and a clinician who screened and initiated clients on PrEP. Screening included HIV testing, hepatitis B screening, renal function testing using a serum creatinine threshold of ≥60 mL/min. and reporting symptoms of sexually transmitted infections (STIs). HIV‐positive individuals were linked to HIV clinics, and individuals with sub‐optimal kidney function and Hepatitis B were linked to regional referral hospitals for further management and not started on PrEP. Syndromic treatment for STIs was offered to symptomatic persons. Clients eligible for PrEP were offered counselling, including the need for daily dosing, side effects and when PrEP can be stopped. They were also given contact information for further questions. Clients were asked to return to clinics at one, three, six, nine and twelve months after PrEP enrolment for refills, adherence counselling, HIV retesting, assessment of HIV risk (including STI screening) and side effects.. However, refill schedules were flexible depending on client preferences. Phone calls were done for clients who missed their visit and had provided phone contact. Client peers and members of village health teams were engaged to find clients who had no phone contacts. Client peers included sex workers and fisher folk using PrEP. Community Retention in the programme was defined as returning for a scheduled visit and getting a refill of PrEP. Clients who did not return for their PrEP refills were assumed to have discontinued PrEP since PrEP was only available through RHSP in south‐central Uganda. PrEP uptake was defined as starting PrEP within one month of screening for eligibility.

### 2.2 Statistical methods

The study used secondary data from registers of the PrEP programme. All the data available at implementing sites from April 2018 to March 2019 were used in the analysis. We conducted descriptive analysis of the PrEP cascade estimating the proportions of screened clients eligible for PrEP, the proportion of eligible clients for whom PrEP was contra‐indicated, the proportion of eligible clients who initiated PrEP and the proportion who discontinued PrEP. Multivariable modified Poisson regression [14, 15] was used to estimate adjusted prevalence ratios (aPR) and 95 percent confidence intervals (CI) of PrEP uptake by baseline characteristics including age, marital status, risk category (fisher folk, sex workers, discordant couples, truck drivers and others at substantial risk of HIV relative to HIV‐negative partners in discordant relationships), stratified by sex. We used Kaplan–Meier survival analysis to evaluate retention on PrEP at one, three, six, nine and twelve months following PrEP enrolment with a window of up to 4‐4 weeks after the visit, and log‐rank tests to assess cumulative differentials in survival functions by sex. Clients who were seen within four weeks after a visit were considered retained at the respective scheduled visit. Clients were considered as retained if they returned for their PrEP refills. Multivariable Cox regression was used to estimate adjusted relative hazards (aRH) and 95% CIs for the association between covariates and PrEP discontinuation. Two‐sided tests at 5% alpha were used for statistical inference.

### 2.3 Ethical considerations

The programme evaluation was approved by the Uganda Virus Research Institute Research and Ethics Committee, the Uganda National Council for Science and Technology and the Johns Hopkins University School of Medicine Institutional Review Boards (IRBs). It was also reviewed in accordance with the Centers for Disease Control and Prevention (CDC) human research protection procedures and was determined to be research, but CDC investigators did not interact with human subjects or have access to identifiable data or specimens for research purposes. Individual consent to use clients’ secondary data was waived by the IRBs.

## 3 RESULTS AND DISCUSSION

### 3.1 Screening and uptake of PrEP

**Figure 1:**
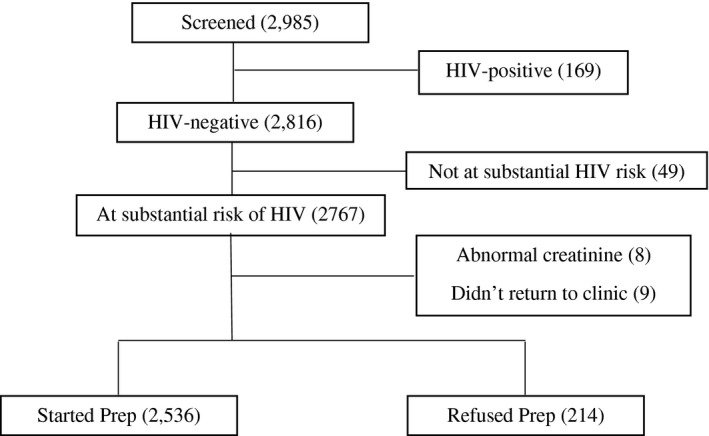
Enrolment Schema for study participants screened for PrEP program in Rakai and neighboring districts, 2018/2019

Of the 2,985 individuals screened for PrEP, 169 (5.6%) were HIV‐positive. Of the 2816 HIV‐negative individuals, 2,767 (98.3 %) were at substantial HIV risk and 2,750 (99.4%) were offered PrEP (after excluding 8 [0.3%]) with sub‐optimal kidney function and 9 who did not return to the clinic after the initial assessment). Of the 2750 individuals offered PrEP, 2,536 (92.2%) accepted and were enrolled (Figure 1). Table 1 shows the characteristics of those enrolled. Among men, enrolees were mainly fisher folk (48.3%) and truck drivers (36.4%), and approximately 52% were married. Most women were sex workers (82.8%), and 20.0% were married. Most clients were aged 20 to 29 (men 48%, women 55.8%).

**Table 1:** Distribution of baseline characteristics of clients enrolled in the pre‐exposure prophylaxis (PrEP) programme in Rakai, Uganda and neighbouring districts (2018 to 2019)

	Women (N = 1608)		Men (N = 928)		Total (N = 2536)	
	Number/mean	Percent/SD	Number/mean	Percent/SD	Number/mean	Percent SD
Age, mean/SD (years)	25.8	7.3	29.8	9.2	27.2	8.2
Age, group (years)						
15 to 19	299	18.6	79	8.5	378	14.9
20 to 24	574	35.7	235	25.3	809	31.9
25 to 29	324	20.1	211	22.7	535	21.1
30 to 34	180	11.2	173	18.6	353	13.9
≥35	231	14.4	230	24.9	461	18.2
Marital status						
Married	322	20.0	485	52.3	807	31.8
Separated/divorced	670	41.7	136	14.6	806	31.8
Single	616	38.3	307	33.1	923	36.4
Category						
HIV‐discordant couples	106	6.6	95	10.2	201	7.9
Fisher folk	80	5.0	448	48.3	528	20.8
Other	90	5.6	47	5.1	137	5.5
Sex workers	1332	82.8	–	–	1332	52.5
Truck drivers	–	–	338	36.4	338	13.3

### 3.2 Acceptance and retention on PrEP

Fisher folk were less likely to accept PrEP compared to HIV‐discordant couples (men, aPR = 0.91 [95% CI = 0.85 to 0.97]; women, aPR = 0.85 [95% CI = 0.77 to 0.94]). Acceptance did not differ significantly by age or marital status (Table 2).

**Table 2:** Prevalence ratios (PR) of pre‐exposure prophylaxis (PrEP) uptake and relative hazards of discontinuation of PrEP associated with covariates among men and women in Rakai, Uganda and neighbouring districts (2018 to 2019)

	Women						Men					
	Number accepted PrEP/number eligible	Percent	Adjusted acceptance prevalence ratios (95% CIs)		Adjusted discontinuation relative hazards (95% CI)		Number accepted PrEP/number eligible	Percent	Adjusted acceptance prevalence ratios (95% CIs)		Adjusted discontinuation relative hazard (95% CI)	
			aPR^a^	95% CI	aRH^a^	95% CI		%	aPR^a^	95% CI	aRH^a^	95% CI
All	1608/1729	93.0	–	–	–	–	928/1021	90.9	–	–	–	–
Age groups												
15 to 19	299/323	92.6	1		1		79/89	88.8	1		1	
20 to 24	574/619	92.7	0.99	0.95 to 1.04	0.92	0.79 to 1.07	235/260	90.4	1.01	0.93 to 1.09	0.86	0.66 to 1.12
25 to 29	324/347	93.4	1.01	0.96 to 1.06	0.88	0.73 to 1.05	211/229	92.1	1.04	0.96 to 1.13	0.87	0.66 to 1.15
30 to 34	180/195	92.3	1.01	0.95 to 1.06	0.78	0.63 to 0.96	173/191	90.6	1.04	0.95 to 1.13	0.81	0.60 to 1.08
35+	231/245	94.3	1.02	0.97 to 1.08	0.83	0.68 to 1.03	230/252	91.3	1.05	0.97 to 1.15	0.82	0.61 to 1.10
Marital status												
Married	322/347	92.8	1		1	–	439/485	90.5	1		1	
Separated	670/714	93.8	0.99	0.95 to 1.04	0.97	0.82 to 1.14	123/136	90.4	1.02	0.96 to 1.08	1.09	0.89 to 1.32
Single	616/668	92.2	0.98	0.94 to 1.03	0.90	0.76 to 1.08	281/307	91.5	1.03	0.98 to 1.07	1.07	0.90 to 1.28
Category												
Discordant couples	106/110	96.4	1	–	1	–	95/100	95.0	1	–	1	–
Fisher folk	80/98	81.6	0.85	0.77 to 0.94	1.99	1.46 to 2.72	448/520	86.1	0.91	0.85 to 0.97	1.37	1.07 to 1.76
Sex workers	1332/1419	93.9	0.99	0.94 to 1.04	1.42	1.09 to 1.83	–	–	–	–	–	–
Truck drivers	–	–	–	–	–	–	338/345	98.0	1.04	0.98 to 1.10	1.49	1.14 to 1.94
Other	90/102	88.2	0.95	0.87 to 1.03	1.74	1.25 to 2.41	47/56	83.9	0.92	0.82 to 1.03	1.39	0.97 to 2.04

^a^ Analyses were adjusted for baseline characteristics including age, marital status and risk category (fisher folk, sex workers, discordant couples, truck drivers and others at substantial risk of HIV relative to HIV‐negative partners in discordant relationships) and stratified by sex.

**Figure 2:**
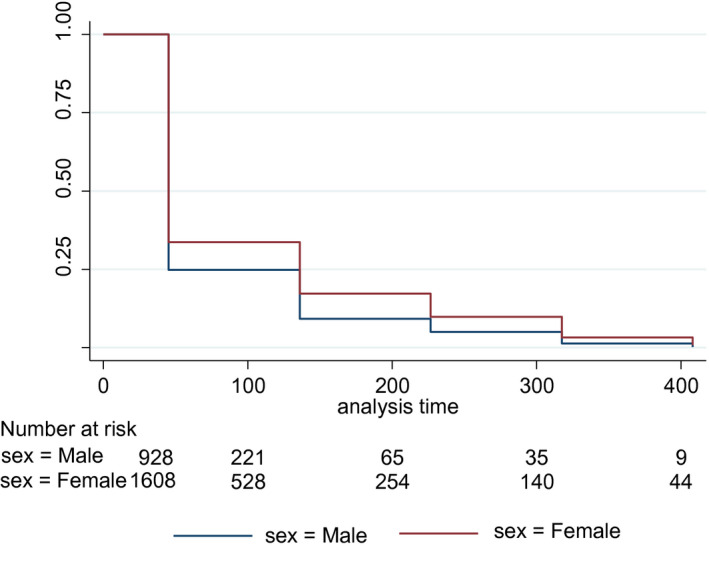
Kaplan‐Meier survival curve for retention of clients on PrEP in Rakai and neighboring districts, 2018/2019

Median retention was 45.4 days for both men and women, but overall retention was higher among women than men (log‐rank, *p* < 0.001, Figure 2). Compared to women in HIV‐discordant couples, sex workers (aRH = 1.42 [95% CI = 1.09 to 1.83]), female fisher folk (aRH = 1.99 [ 95% CI = 1.46 to 2.72]) and women in the “Other” category (aRH = 1.74 [95% CI = 1.25 to 2.41]) were more likely to discontinue PrEP. Compared to men in discordant couples, male fisher folk (aRH = 1.37 [95% CI = 1.07 to 1.76]) and truck drivers (aRH = 1.49 [95% CI = 1.14 to 1.94]) were more likely to discontinue PrEP. The rates of discontinuation tended to decrease with age for both men and women (*p* for trend = 0.001 for men and < 0.001 for women). However, in the multivariable analysis this relationship was only statistically significant for the comparison between women 30 to 34 years and adolescents 15 to 19 years (aRH = 0.78 [95% CI = 0.63 to 0.96]). (Table 2).

## 4 DISCUSSION

We found high initial PrEP uptake rates, consistent with other PrEP implementation studies [9, 10, 11]. PrEP uptake rates were the highest among HIV‐negative partners in HIV‐discordant relationships. However, we found low retention rates especially among sex workers, truck drivers and fisher folk; consistent with earlier studies which showed that mobility and sex work were barriers to PrEP adherence [11, 16]. Flexible programmes tailored to highly mobile sub‐populations could help improve PrEP adherence rates.

Our evaluation has several limitations. We could not determine reasons for PrEP discontinuation (such as low perceived risk due to fewer risk behaviours) because Clients did not return to the clinics and were not actively tracked by the programme. In earlier studies, low perceived risk was linked to low PrEP adherence [17]. To establish reasons for discontinuation, future PrEP scale‐up efforts could consider tracking of clients who drop out of programmes; using approaches such as short messaging services which, in some studies, were shown to be acceptable and preferable to in‐person visits [18]. Such tracking could help estimate the number of individuals who may no longer need PrEP or who used other services. This is consistent with the proposed prevention‐effective approach in which individuals may discontinue PrEP when they do not feel at risk owing to adoption of other prevention strategies or changes in their HIV risk [19, 20]. In our upcoming qualitative publications, we will provide information on reasons for discontinuation.

The programme did not assess client preferences or perceived stigma associated with PrEP use. Studies in Kenya and Malawi showed that sex workers valued confidentiality, privacy, and trustworthiness [6] and preferred male providers and non‐stigmatizing locations for drug refills such as family planning clinics or NGO drop‐in centres [21]; suggesting a need to assess client preferences for PrEP refill locations.

In addition, the requirement for clients to return for regular HIV testing as a condition for continued PrEP prescription may have discouraged clients from continuing PrEP as reported in a Kenyan PrEP demonstration project and could be alleviated by HIV‐self testing by PrEP clients between clinic visits [22].

The rates of discontinuation tended to decrease with age. However, this relationship was only statistically significant for comparison between women 30 to 34 years and 15 to 19 years. We did not have enough statistical power to show similar differences between clients 15 to 19 years and other older age groups. We did not have data on refusal of screening, so we cannot comment on the extent to which failure to account for refusal of screening over‐estimates the PrEP uptake at a population level. Our list of predictors of acceptance and retention on PrEP was limited to those collected on programme tools, we therefore cannot rule out residual confounding. Additional variables such as knowledge of partner's status, past HIV testing history, changes in sexual partnerships over time and perceived risk of HIV will be helpful for future research.

## 5 CONCLUSIONS

Uptake of PrEP was high in this population but modestly lower among fisher folk. However, retention rates were low, especially among highly mobile populations and tended to be lower among younger clients. Interventions, distribution systems and tracking mechanisms to optimize PrEP retention for mobile populations and young people are urgently needed.

## COMPETING INTEREST

The authors declare that they have no competing interests.

## AUTHORS’ CONTRIBUTIONS

JK contributed to conception, design, analysis, interpretation, drafting and revision of manuscript. HN contributed to data analysis and interpretation. JB, GN and BK, contributed to acquisition of the data, interpretation and revision of manuscript. SS, AME, LWC, RG, SJR, PK, SA and DS contributed to conception, design, interpretation and revision of manuscript. All authors have read and approved the final version of the manuscript.
